# Accuracy and cost-effectiveness of the INDDEX24 Dietary Assessment Platform in Viet Nam

**DOI:** 10.1017/S0007114522001507

**Published:** 2023-05-28

**Authors:** Jennifer Coates, Winnie Bell, Peter Bakun, Katherine P. Adams, Jérome W. Somé, Brooke Colaiezzi, Ha Thi Phuong Do, Beatrice Rogers

**Affiliations:** 1 Tufts University, Gerald J. and Dorothy R. Friedman School of Nutrition Science and Policy, Boston, MA, USA; 2 Department of Nutrition, Institute for Global Nutrition, University of California, Davis, CA, USA; 3 Institut de Recherche en Sciences de la Santé, Ouagadougou, Burkina Faso; 4 Viet Nam National Institute of Nutrition, Hà Nội, Viet Nam

**Keywords:** Dietary, Global, Assessment, Validation, Technology

## Abstract

Technology-enabled approaches to conducting 24-h dietary recalls (24HR) may reduce dietary assessment bottlenecks in low-resource settings. However, few studies have assessed their performance relative to conventional pen-and-paper interview (PAPI) approaches and none have validated performance against a benchmark (e.g. weighed food record (WFR)) in a low- and middle-income country (LMIC). This study assessed relative accuracy and cost-effectiveness of INDDEX24, a technology-enabled approach to conducting 24HR, compared with a PAPI approach and against an observer WFR. Women aged 18–49 years from northern Viet Nam (*n* 234) were randomly assigned to be interviewed using INDDEX24 or PAPI 24HR following a WFR. The two one-sided *t* test approach assessed the equivalence of each recall modality to the benchmark. Difference-in-differences analysis compared the recall-benchmark results across modalities. Cost per percentage point of accuracy for INDDEX24 and PAPI was derived from accuracy results and the cost to conduct the 24HR. The PAPI and INDDEX24 24HR were statistically equivalent to the WFR for all nutrients except vitamin A. INDDEX24 diverged significantly less than PAPI from the WFR for Fe (0·9 *v.* −1·3 mg) and PAPI diverged less for protein (–3·7 *v.* 7·9 g). At the individual level, 26 % of PAPI and 32 % of INDDEX24 respondents had energy intakes within +/– 10 % of the WFR. INDDEX24 cost $111 004 and the PAPI cost $120 483 (USD 2019), making INDDEX24 more cost-effective across most indicators. INDDEX24 was an accurate and cost-effective method for assessing dietary intake in the study context and represents a preferred alternative to PAPI 24HR in Viet Nam and other LMIC.

Many low- and middle-income countries (LMIC) face a double burden of malnutrition, where undernutrition coexists alongside overweight and obesity along with increasing rates of non-communicable disease stemming from dietary risk factors^(1,2)^. In order to address these challenges, countries must monitor how the food system interacts with dietary patterns and health outcomes and how these forces trend over time^(3–5)^.

Despite the need for dietary data to inform a wide array of food policies and programmes, many LMIC collect nationally representative, quantitative dietary data only rarely, if at all. According to the FAO/WHO GIFT dietary survey inventory, there have been just nine national surveys in Africa since 1981 and just four since 2005^(6)^. One major constraint to more frequent dietary assessment in LMIC is the lack of investment in public research infrastructure, that is, the databases, technological tools and standards needed to facilitate high-quality dietary research for societal benefit.

The USA, Europe and other high-income countries have developed 24-h dietary recall (24HR) software that can be administered via the web or respondents’ mobile phones and links directly to robust food composition databases^(7–13)^. Evidence from high-income countries has shown that digital survey technology can mitigate a number of problems related to accuracy, respondent and researcher burden, monetary and time costs of data collection, efficiency and ease of data coding and analysis, and respondent participation rates^(12,14)^. Due largely to resource constraints for research infrastructure development, many LMIC continue to rely on a system of pen-and-paper interview (PAPI) questionnaires and manual data entry, rather than technology-enabled alternatives. Survey tools and dietary reference data are often assembled ‘from scratch’ for each episodic survey, requiring significant advance preparation before a survey as well as extreme post-survey lags in generating useable results^(15)^. Technology-enabled infrastructure, including computer-assisted personal interview (CAPI) applications and linked electronic databases of dietary reference data, such as food composition and portion conversions, has the potential to improve accuracy and reduce the time and cost of dietary assessments in LMIC.

As one potential solution, the International Dietary Data Expansion (INDDEX) Project developed the technology-enabled INDDEX24 Dietary Assessment Platform, designed to streamline dietary assessment in LMIC^(4)^. INDDEX24 is comprised of a (1) mobile CAPI application (INDDEX24 Mobile App) for collecting individual-level 24HR data on a smartphone or tablet via an interviewer-administered questionnaire, (2) linked web application, called the Global Food Matters Database (FMDB), where food composition, conversion factors, and other dietary reference data are stored, publicly shared, and integrated with the INDDEX24 Mobile App at multiple stages of the survey process, and (3) linked analytical reporting feature to facilitate data processing and analysis^(4)^.

Though technological solutions offer great promise, rigorously designed validation studies are needed to determine whether a technology-enabled approach to 24HR surveys is at least as accurate and cost-effective as the PAPI ‘standard of practice’ in LMIC. A limited number of studies have examined the potential benefits of a CAPI approach to 24HR data collection in LMIC, without formal validation^(16,17)^. We identified two studies from LMIC that compared a CAPI with a PAPI modality of 24HR administration^(18,19)^. To our knowledge, no study in a LMIC context has compared CAPI (or a more elaborate ‘technology-enabled’ platform) and PAPI modalities of administering a 24HR survey with a benchmark method such as a weighed food record (WFR). Likewise, no validation study in a LMIC setting has yet examined the relative cost and cost-effectiveness of different dietary recall modalities, including whether a comprehensive technology-enabled solution comprised of CAPI and a synchronised dietary reference database reduces the time and cost of data collection, processing and analysis.

This study, implemented by the Tufts University Friedman School and the National Institute of Nutrition (NIN) in Viet Nam, alongside a parallel effort by the INDDEX Project in Burkina Faso^(20)^, aims to fill the following knowledge gaps in the context of Viet Nam: (1) What is the relative accuracy of INDDEX24 compared with a PAPI 24HR for measuring intake of specific food groups and nutrients of public health concern in LMIC? (‘accuracy component’) (2) What is the relative cost-effectiveness of the INDDEX24 *v*. PAPI modality (‘cost-effectivenes component’), defined as cost-per-average percentage point of accuracy? The results are intended to guide LMIC governments and researchers in choosing the dietary assessment approach that offers the greatest returns on accuracy and cost in resource-constrained contexts.

## Subjects and Methods

### Study design and sample selection

**Fig. 1.**
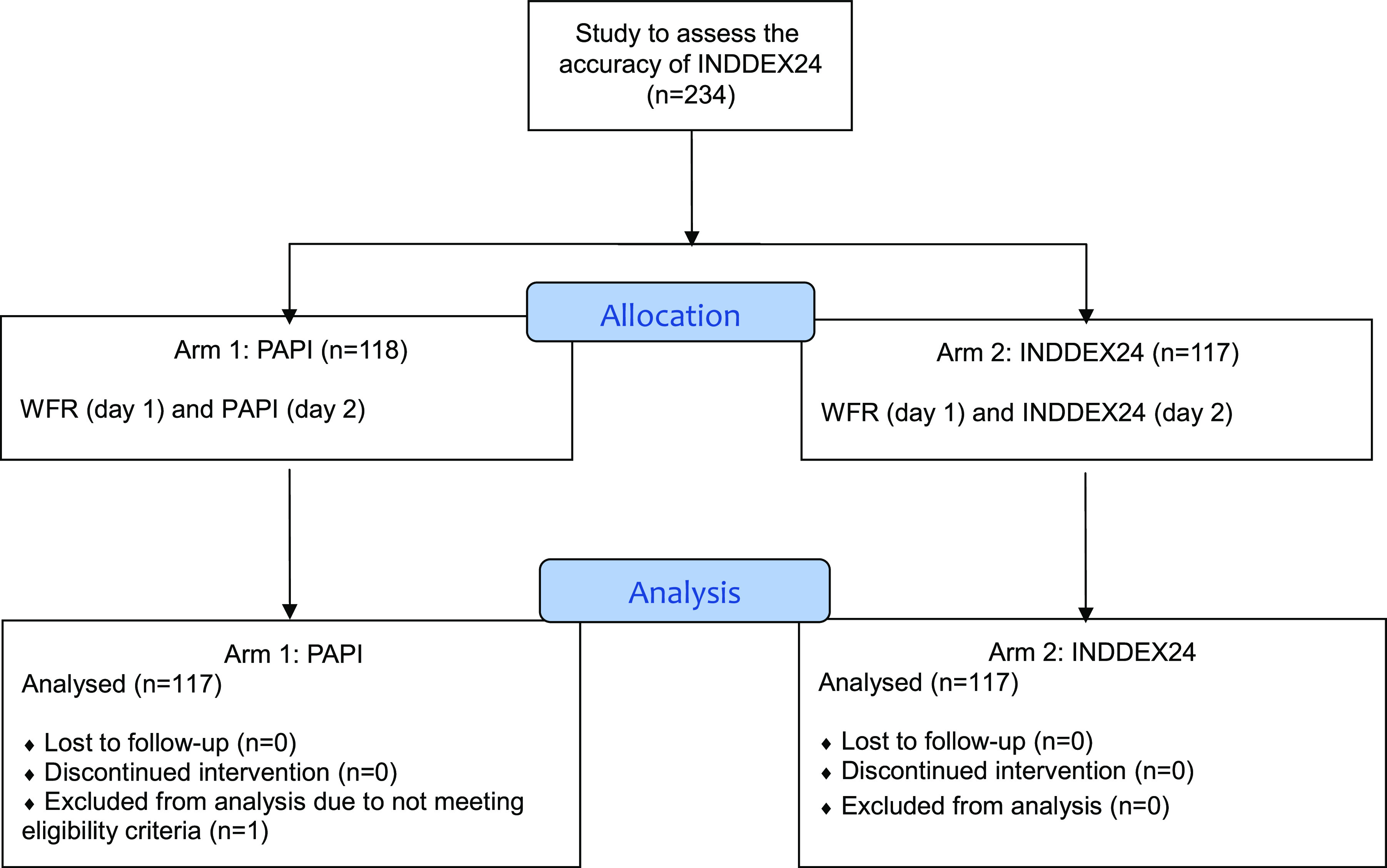
Study design for INDDEX24 validation study in Viet Nam: accuracy component. PAPI, pen-and-paper interview; INDDEX, International Dietary Data Expansion; WFR, weighed food record

**Fig. 2.**
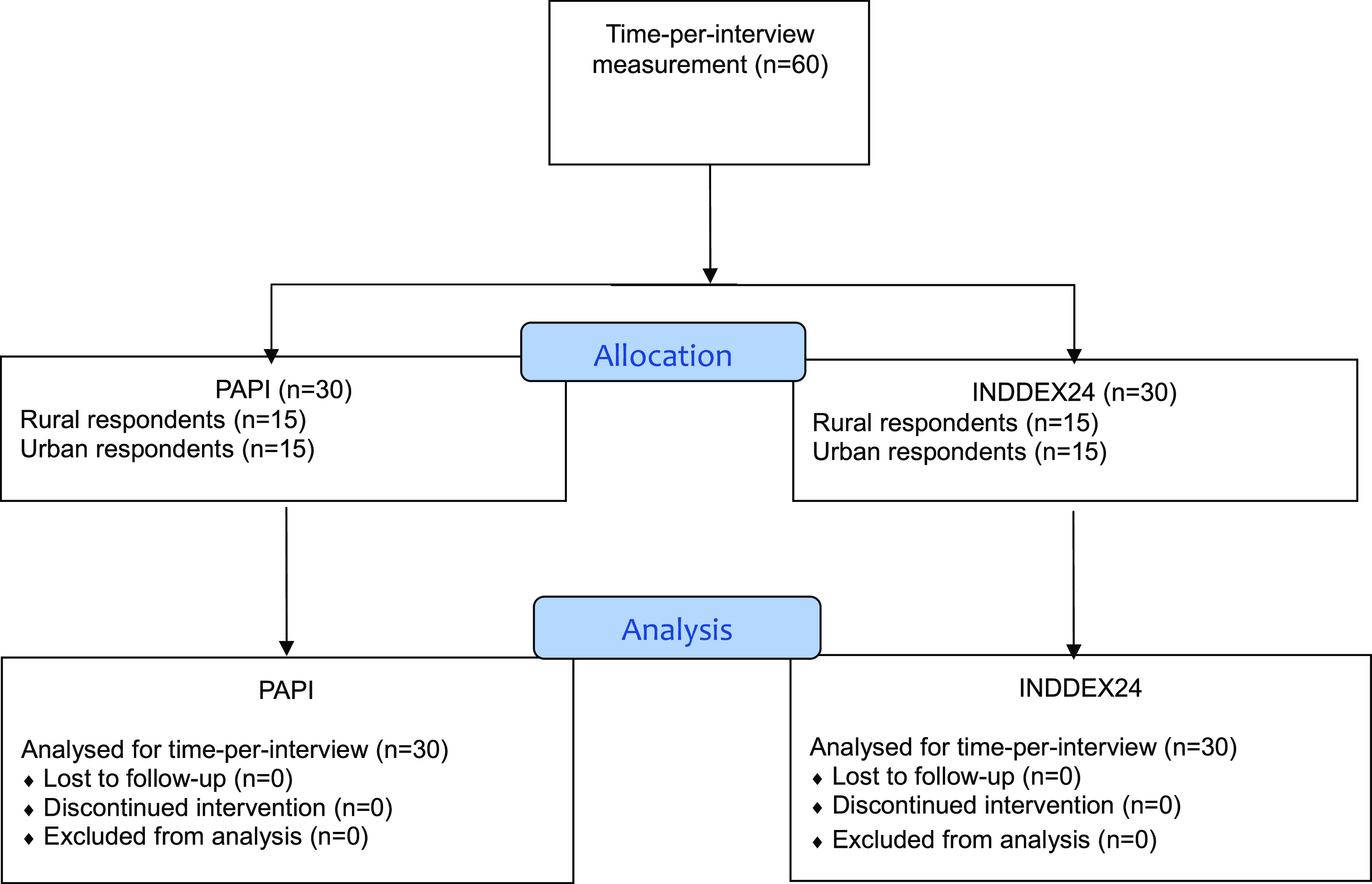
Study design for INDDEX24 validation study in Viet Nam: time-per-interview measurement for cost-effectiveness component. INDDEX, International Dietary Data Expansion; PAPI, pen-and-paper interview

Participants in the accuracy study component (Fig. 1) were recruited to undergo a full-day observed WFR, followed by a randomly allocated INDDEX24 or PAPI 24HR the following day. A separate sample of respondents participated in a 24HR with either INDDEX24 or PAPI only (no WFR), in order to yield a ‘naïve’ estimate of time-per-interview to inform the cost and cost-effectiveness study component (Fig. 2).

The sample size for the accuracy component was calculated based on Pearson’s correlation tests for the strength of association between measurements of the test modalities (INDDEX24 and PAPI) and the reference method (WFR). A correlation below 0·6 between the WFR-INDDEX24 and WFR-PAPI was considered unsatisfactory, whereas an increase in correlation of 0·15 or greater (correlation 0·75 or greater) was considered a practically important improvement. *α* and *ß* were set at 5 % and 80 %, respectively. Given these parameters, the sample size was estimated at *n* 104 for each arm and increased to *n* 117 to account for an estimated 12 % non-response (*n* 234).

The study sites were situated in the Thanh Oai and Hai Ba Trung Districts of Hanoi Province in the Red River Delta region, located in northern Viet Nam. NIN selected the study sites purposively for variability in urbanicity, logistical convenience and socio-demographic characteristics typical of the range of income, livelihoods and housing found in northern Viet Nam. Hanoi Province has some of the lowest poverty in the country. About 33 % of the employed population works in agriculture, 23 % in non-farm self-employment and 44 % in wage work^(21)^. Thanh Oai District (accuracy and time-per-interview components) is located about 25 km southwest of the capital city, Hanoi. Though administratively classified as ‘rural’, given its proximity to Hanoi and the extent of migration to and from the city for day labour, some areas have peri-urban characteristics. Hai Ba Trung District (time-per-interview component), in contrast, is in the bustling centre of Hanoi.

For the accuracy study component, 234 eligible women aged 18–49 years (maximum one per household) were randomly selected from a list-based sampling frame of all female residents of six randomly selected communes (i.e. subdistricts) in Thanh Oai District. The sampling frame was provided to NIN for this study by the rural Thanh Oai District community health services. The study coordinator selected respondents from the list using the Excel RAND function. Sampled women (*n* 234) were allocated to either (1) PAPI and WFR (*n* 117) or (2) INDDEX24 and WFR (*n* 117) through the random assignment, to respondents, of enumerators who alternated between conducting a 24HR with INDDEX24 or PAPI every other day.

To inform the cost-effectiveness study component, time-per-interview was assessed by administering the PAPI and INDDEX24 24HR to sixty women aged 18–49 years who were not exposed to WFR prior to completing a 24HR. The sample was stratified between one randomly selected subdistrict in Thanh Oai District (*n* 30) that was not part of the accuracy study and one randomly chosen subdistrict in Hai Ba Trung District (*n* 30). As with the accuracy component, the time-per-interview sample was drawn from a list-based sample frame maintained by district health representatives of these two districts. Enumerators were randomly assigned to each respondent and alternated every other day between 24HR modalities. Half of the thirty respondents received the PAPI and half received the INDDEX24 24HR in each district.

Community health workers and one staff member from NIN contacted sampled respondents in early August 2019, before data collection began, in order to explain the objectives of the study and obtain informed consent. The protocol stipulated that potential respondents be excluded based either on noticeably poor health that could interfere with their ability to be observed and interviewed (e.g. dementia and visible illness) or on an unwillingness to participate in both the WFR and 24HR components of the study. One respondent was replaced for not meeting the eligibility criteria.

### Dietary assessment methods

#### Weighed food record

The observed WFR was selected as the benchmark method in this validation study as it is considered to provide an accurate estimate of individual consumption^(22,23)^. This method typically requires an enumerator to be present in the household throughout the day for all meal preparation and consumption periods (and accompany the respondent if she leaves the house). Using this method, the enumerator records detailed, real-time information on all foods and beverages prepared and consumed, including recipes, ingredients (henceforth referred to as ‘foods’ or ‘food items’), and their weights. The WFR instrument was developed and pilot-tested before the start of data collection and consisted of three paper forms: a registration form, a recipe form to capture all household foods prepared and a consumption form to capture foods eaten.

#### INDDEX24 and pen-and-paper interview individual quantitative 24-h dietary recall dietary recall

The multiple-pass 24HR method was used for both test modalities (INDDEX24 and PAPI) based on the version developed and validated by Gibson and Ferguson for use in LMIC^(24,25)^. Prior to the validation study, the INDDEX24 Dietary Assessment Platform was rigorously developed and tested over a period of 4 years, guided by the Tufts INDDEX team and a technical advisory committee of globally recognised dietary assessment experts, with significant input from partners in both Viet Nam and Burkina Faso. A feasibility study was carried out in both countries in 2018 in order to assess the usability and usefulness of the initial version of the INDDEX24 Dietary Assessment Platform. Respondents offered feedback through cognitive debriefing, and enumerators and technical experts (in both Viet Nam and Burkina Faso) were engaged through focus group discussions^(26)^. The findings were positive, and most respondents found INDDEX24 quicker, easier and more convenient than a PAPI 24HR^(26)^. Enumerators also offered detailed suggestions for improvements to the INDDEX24 Mobile App interface and structure that were incorporated into v2.1 of the INDDEX24 Dietary Assessment Platform that was used for the accuracy validation study.

The PAPI arm of the validation study was a 24HR paper data collection instrument that was adapted to be as similar to the INDDEX24 instrument as possible. Both INDDEX24 and PAPI followed the 24HR multiple-pass technique; however, there were some notable differences by modality. When conducting an INDDEX24 24HR, after entering foods, time and place of consumption in the First Pass (i.e. the ‘quick list’), enumerators would move to the Second Pass and select the food or standard recipe from the pre-defined dropdown list after probing for more details, per the instructions in the app. Any foods that were consumed more than once could be selected and copied from the respondent’s food list, rather than going back to the master list. Following the selection of the food or recipe from the list (or inputting a missing food item or non-standard recipe (NSR) name), the portion size was then estimated in the Third Pass. If a recipe did not exist in the INDDEX24 database, then the enumerator would record all the details as a NSR by either copying and modifying a standard recipe or by entering a new recipe including total cooked weight and the quantity of each ingredient used in the NSR Pass. The Fourth Pass was meant for reviewing a summary of the interview, in which a summary screen provided an overview of all foods, quantities and approximate energy consumed. As additional foods for the food list were identified during piloting, training and data collection, the updated master INDDEX24 food list and conversion factors in the FMDB were updated onto all enumerator tablets via wireless connection.

In contrast to the INDDEX24 24HR, during the PAPI 24HR, enumerators followed the same four passes but used a printed food list that contained all of the same items as those added to the INDDEX24 food list up to the point where training ended (i.e. no new items were added to the PAPI printed version after the start of data collection, reflecting a real-life constraint of working with a PAPI approach). The enumerators recorded details on each food and then searched the printed food list for the corresponding item and code. If the enumerator could not find a food or recipe, they would describe it in detail and code it as ‘99 999’. If a standard recipe did not exist that matched what was reported by the respondent, then the enumerator recorded it as a NSR and collected detailed information about the amount prepared and the quantities of ingredients used. Unlike with INDDEX24, which pre-assigned portion size estimation aids (PSEA) and probes to foods, when enumerators were administering the PAPI, they were expected to identify which would be the best PSEA option for each food and probe to find the most accurate match.

### Preparation of dietary reference data

The dietary reference data for the study consisted of a list of over 3000 foods, standard recipes and ingredients (henceforth ‘food list’), as well as portion conversions, yield factors, retention factors and food-specific survey probes. The dietary reference data were developed for this study as well as for the 2019 Viet Nam National Dietary Survey^(27)^. The food list was collated from a variety of data sources, including the published Viet Nam Food Composition Table^(28)^, NIN Food Photo Atlas^(29)^, NIN recipe book ‘Nutritive Values of 500 Common Dishes’^(30)^ (based on household recipes reported in the 2010 General Nutrition Survey), the NIN yield factors book with simple cooked foods^(31)^, the INDDEX Project Feasibility Study (*n* 60) conducted in 2018 and the Nutritive Value of Common Street Foods with street food recipes from southern Viet Nam^(32)^. Standard recipes for mixed dishes were calculated based on the methods described by Gibson and Ferguson (2008)^(24)^.

The items in the food list were matched primarily with the Viet Nam NIN Food Composition Table^(28)^, and other FCT were selected based on the availability of the data and the similarity of the food or country of origin as advised by FAO^(33–40)^. The 2017 Vietnamese FCT contained only raw foods. For the purpose of the validation study, the FCT was expanded to include simple cooked foods (e.g. boiled, baked and steamed). For any non-raw foods, adjustments were made with the appropriate cooking yield and nutrient retention factors. All values were checked using FAO/INFOOD guidelines^(41)^.

Portion size estimation methods used for the 24HR included direct weight, a photo atlas, proxy weight (with rice) and proxy weight (with water). In a few select cases where food was purchased outside the home, respondents estimated the price or weight of the food purchased. Portion conversion factors accounted for the density and edible portion, as appropriate. Details about each component of the dietary reference data are available in the Online Supporting Material, Supplement I.

### Training and data collection procedures

A total of twenty-one WFR enumerators and four WFR supervisors were trained for 5 d. For the 24HR, twelve 24HR enumerators and three 24HR supervisors were trained for a total of 10·5 d on both the INDDEX24 and PAPI modalities. Training consisted of classroom learning and field-based practice.

Data collection occurred in August 2019 and was evenly distributed across all 7 d of the week. The WFR took place over the course of a full day, the day prior to the 24HR interview. Enumerators arrived at the respondent’s house in time to observe the preparation and consumption of the first food and drink of the morning and stayed through the last main meal of the evening. WFR enumerators recorded detailed information on food name, food code, quantity prepared, weights of pans and plates, quantity served, and amount leftover, as well as the time and place of preparation and consumption for each item. For meals where the foods were eaten communally, the WFR enumerators asked respondents to apportion the respondent’s meal into a second set of serving bowls that the respondent would eat. These serving bowls (e.g. rice bowl, dipping sauce, etc.) were weighed before and after consumption to determine the amount consumed by the respondent. If mixed dishes (i.e. those derived from a recipe) were purchased outside the home, prepared elsewhere (e.g. by a relative or neighbour), or leftover from a dish prepared the previous day, then enumerators recorded as many details as possible, and these dishes were later matched with standard recipes. WFR enumerators coded all items throughout the course of the day in between eating occasions, and codes were later checked by supervisors. Any discrepancies were discussed and addressed between the supervisor and the enumerator.

The team of 24HR enumerators administered the INDDEX24 or PAPI 24HR interview the day after the WFR at the respondent’s house at an appointed time (either in the morning or the afternoon). The 24HR enumerators were assigned to conduct either INDDEX24 or PAPI interviews for the day and alternated modality by day. For both INDDEX24 and PAPI, the enumerator first registered the respondent, then conducted the 24HR followed by the socio-demographic module and self-reported respondent weight.

All food weighing (WFR, INDDEX24 and PAPI) was performed with digital scales (My Weigh KD 7000 7kg, accurate within 1g, that were calibrated by the supervisors every day using a standard weight). In addition, each time the scale was moved in the course of the day, the enumerator checked the scale with a 100-g reference weight afterwards to ensure that it was still accurate. Data collection for the time-per-interview component also occurred in August 2019. The approach for the INDDEX24 and PAPI interviews followed the same structure as described above for the accuracy portion of the study, except that the WFR was not conducted in this component. With INDDEX24, the time-per-interview was recorded automatically by the mobile app, yielding the time per pass and for the total interview as part of the master data file. For the PAPI 24HR, time-per-pass and time-per-interview were collected manually by each enumerator using a phone stopwatch and recorded on a paper form designed for this purpose.

This study was conducted according to the guidelines laid down in the Declaration of Helsinki, and all procedures involving human subjects were approved by the institutional review boards at Tufts University (#1 904 024) and Viet Nam NIN (45/VDD-QLKH). Written informed consent was obtained from all subjects. Participants in the accuracy component of the study received approximately 25 USD paid in VND (17 USD for the WFR day and 8 USD for responding to the 24HR) to help offset the cost of their time. Participants in the time-per-interview component who responded to the 24HR received the equivalent of 8 USD.

### Data entry, cleaning and processing: accuracy component

For INDDEX24, no additional data entry was required after enumerators recorded data in the mobile app at the interview site. Matches with appropriate conversion factors, food codes and nutrients were made automatically through the INDDEX24 analytical reporting function. Dietary reference data for foods, NSR (including food away from home and food prepared on the previous day) or PSEA conversion factors that were reported in the survey but did not already exist in the INDDEX24 FMDB were subsequently added to the FMDB before analysis.

All WFR and PAPI data were double-entered using CSPro7.3^(42)^ with data entry forms developed for the purpose of this study. Two separate sets of data entry clerks were trained to enter the data. A data entry supervisor resolved discrepancies between the double-entered data by consulting the hard copies of the questionnaires. Subsequent cleaning was carried out to ensure the appropriate use of food, recipe, and ingredient codes, PSEA codes, and household ID.

For both the WFR and PAPI, all foods and recipes were converted to nutrient values by manually matching with INDDEX24 FMDB dietary reference data that were entered into the CSPro database developed to house the WFR and PAPI data. Any new foods, NSR (including food away from home and food prepared on the previous day) or PSEA conversion factors that did not already exist in the INDDEX24 dietary reference dataset were added to the CSPro database to ensure that they had a match with the WFR and/or PAPI.

Given that the WFR recorded data from the first meal to the last meal of the day but the 24HR asked respondents to recall all foods consumed in the previous 24 h, the 24HR analysis was bounded to match the time of the observed WFR. For the 24HR, the respondents were asked to report the approximate time of consumption but may not have reported the exact time, so a 45-min buffer was added to either side of the WFR to ensure inclusion of all 24HR start and end times. This resulted in the inclusion of more than 95 % of all food items across the 24HR (96·27 % for INDDEX24 and 95·70 % for PAPI). On average, the recall window was from 06.38 (35 m sd) to 19.03 (1 h 14 m sd) for INDDEX24 and 06.44 (33m sd) to 19.09 (1 h 51 m sd) for the PAPI.

The following nutrients of public health concern in many LMIC were selected for analysis: energy (kcals), fat (g), protein (g), carbohydrates (g), total fibre (g), vitamin A (mcg retinol activity equivalent), vitamin C (mg), Ca (mg), Fe (mg) and Zn (mg). To better approximate a normal distribution, nutrients were log-transformed. All benchmark (WFR) daily intakes for both groups fell within a standard cut-off of 500–5000 kcal and were kept for analysis regardless of their recall values^(43–45)^.

Despite extensive efforts to update the FCT, it remained incomplete for certain nutrients. Of the foods reported in the validation study for our nutrients of interest, 97·0 % had energy values, 92·5 % had protein values, 82·9 % had fat values, 92·5 % had carbohydrate values, 82·1 % had fibre values, 90·0 % had vitamin A retinol activity equivalent values, 79·7 % had vitamin C values, 88·5 % had Ca values, 85·3 % had Fe values and 69·0 % had Zn values. The distribution of missing FCT values for each nutrient was equivalent across the INDDEX24 and PAPI modalities and thus does not affect the results of the study, as the research questions centre on relative validity and not absolute intake.

### Data analysis

The primary objective of this study was to measure the differences between INDDEX24 and WFR, the PAPI 24HR and WFR, and the difference-in-differences between the two recall modalities. As a first step, Bland–Altman plots^(46,47)^ were generated for grams consumed and for consumption of energy and all nutrients of interest in order to assess visually the level of agreement between each 24HR modality and the WFR. Next, following Arsenault *et al.* ^(48)^, the two one-sided *t* test approach was used, pairing each individual’s recall and WFR on all nutrients of interest. The two one-sided *t* test is a paired sample comparison of means. Unlike difference testing, it evaluates whether the difference between the two collection methods is within a pre-specified range and aims to demonstrate equivalence by way of the lack of difference:

H0: (µ2–µ1) ≤ –*ϵ* or (µ2–µ1) ≥ *ϵ*;

H1: –*ϵ* < (µ2–µ1) < *ϵ*;

where (– *ϵ*, *ϵ*) is the pre-specified range of acceptable difference. One test of equivalence is performed on each bound and uses the larger *P*-value to compare *α*. Rejecting the null hypothesis of a sufficiently large difference, the alternative hypothesis of ‘nearly equivalent’ is accepted. *Post hoc* power computation for the two one-sided *t* test using the *n* 234 sample size remained over 80 % for most outcomes tested at a delta range of −0·5 to 0·5.

For log-transformed data such as these, the test uses the lognormal distribution, and therefore the ratio of the geometric mean differences was used as the centre. The standard 10 % bound was chosen for the equivalence buffer^(48–50)^. Once statistical equivalence was established between the WFR and 24HR pairs for both INDDEX24 and PAPI, the magnitude of the difference between the WFR-INDDEX24 and the WFR-PAPI was then assessed using a difference-in-differences approach. In this unadjusted random subjects model, nutrient outcomes were modelled on collection type (WFR and 24HR) and modality (PAPI and INDDEX24), estimating the difference of the WFR and 24HR for each modality and comparing these differences between modalities. SAS 9.4 Proc mixed was used.

To quantify the individual accuracy of the INDDEX24 and PAPI 24HR, the percentage of respondents using each 24HR modality that fell within specific percent error categories compared with the WFR for energy and each nutrient was calculated. This approach assessed the extent to which each modality (INDDEX24 and PAPI) was accurate at the individual level relative to the WFR estimates. All analyses used SAS 9.4 and Stata 15 SE.

### Data analysis: cost-effectiveness component

The cost-effectiveness of each modality was calculated by combining cost data with INDDEX24 and PAPI indicators of food and nutrient intake accuracy, with ‘effectiveness’ defined in terms of percentage points of accuracy gained through each of the two modalities. Cost data were derived from a costing study, reported in detail elsewhere, that used an activity and ingredients-based method to estimate the total and relative costs of each activity needed to complete the 24HR survey and produce a clean, analysable 24HR dataset using the INDDEX24 *v*. PAPI modality^(51)^. The cost study adopted a societal perspective such that all costs were accounted for (including respondent time) regardless of who incurred them^(52)^. Costed activities included the preparation of dietary reference data, survey preparation, training, survey execution (including time-per-interview for both enumerators and respondents), data entry, and data cleaning and processing. The reported time and monetary costs associated with completing each activity were combined with information on staff wages and salaries, converted from Vietnamese Dong to US dollars where necessary, adjusted to 2019 US dollars, and summed in order to estimate the total cost of conducting the 24HR using INDDEX24 and the PAPI modalities.

Cost-effectiveness was assessed based on three primary outcomes: average percent accuracy in estimating the number of food items consumed (item count), average percent accuracy in estimating total gram amount of food intake and a composite measure of the average percent accuracy in estimating each of ten nutrients of interest (energy, fat, protein, carbohydrate, fibre, vitamin A, vitamin C, Ca, Fe and Zn), hereafter referred to as the ‘ten-nutrient composite measure of nutrient intake’. For the number of food items consumed and gram amount, the average percent error was calculated as one minus the ratio of the average estimate based on INDDEX24 (or based on PAPI) to the average estimate based on the WFR, and then percent accuracy was defined as 100 minus the absolute value of the average percent error. For gram amount, for example, the average percent accuracy of INDDEX24 and PAPI was calculated as:

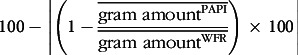




For the ten-nutrient composite measure of nutrient intake, a measure of overall accuracy in estimating nutrient intake, we followed Hatløy, Torheim and Oshaug^(53)^ whose methods have been applied for validation of dietary metrics^(54,55)^. First, the average percent accuracy in estimating the intake of each nutrient was calculated, using the same method as described for item count and gram amount, and then the overall average was taken across the ten nutrients. For each measure of accuracy, absolute and relative (i.e. the difference between INDDEX24 and PAPI), cost-effectiveness was calculated as total cost per average percentage points of accuracy.

## Results

Table 1 compares key socio-demographic characteristics among respondents who completed INDDEX24 and PAPI 24HR. The two groups of respondents were similar for most characteristics, though educational achievement was higher among INDDEX24 respondents, and slightly more PAPI respondents were pregnant and breast-feeding.

**Table 1.** Socio-demographic characteristics of PAPI and INDDEX24 respondents (*n* 234) in Thanh Oai, Viet Nam

	PAPI	INDDEX24
	%	%
Pregnant	5·1	1·7
Breast-feeding	12	9·4
Education level (some/all completed)		
Primary	0·9	0
Secondary	22·2	17·9
High school	30·8	30·8
College (2 years)/university (4 years)	40·2	47·9
No education	0·9	0
Professional training	5·1	3·4
Weekly frequency of food preparation by respondent		
All or most of the time	76·9	71·8
Sometimes (3–6 meals)	13·7	19·7
Rarely (1–2 meals)	9·4	8·5
Main mode of transportation to buy food		
Walking	13·7	10·3
Bicycle	24·8	20·5
Motorbike	59·8	67·5
Other	1·7	1·7

PAPI, pen-and-paper interview; INDDEX, International Dietary Data Expansion.

### Differences in estimates of nutrient intakes by pen-and-paper interview *v.* INDDEX24 24-h dietary recall modalities, compared with weighed food record

The average level of overestimation and underestimation when comparing the INDDEX24 and PAPI 24HR to the WFR was minor with an equivalence bound set at 10 % (Table 2). The two one-sided *t* test indicated statistical equivalence between both 24HR modalities and WFR for energy and all nutrients of interest with the exception of vitamin A, which showed an overestimate for PAPI and a sizable underestimate for INDDEX24.

**Table 2.** Difference-in-difference comparison of group means for energy and nutrients for PAPI and INDDEX24 to WFR in Thanh Oai, Viet Nam (Mean values and standard deviations, *n* 234)

	Arm 1: PAPI (*n* 117)							Arm 2: INDDEX24 (*n* 117)							
	WFR		PAPI		WFR-PAPI			WFR		INDDEX24		WFR-INDDEX24			Arm 1 – Arm 2
	Mean	sd	Mean	sd	Mean difference^a^	sd	Equivalence 10 % bound *n*, *P^b^*	Mean	sd	Mean	sd	Mean difference	sd	Equivalence 10 % bound *n*, *P*	Diff-in-diff *P^c^*
Item count	37·6	11·7	34·5	9·4	3·1	9·3	100, < 0·001	40·0	13·8	36·4	12·3	3·6	8·8	99, < 0·001	0·74
Gram amount	2742·8	780·5	2865·0	894·0	–122·1	736·4	115, < 0·001	2725·4	727·1	2803·9	862·4	–78·5	742·2	117, < 0·001	0·55
Energy (kcal)	1734·5	490·5	1850·4	574·5	–115·9	468·6	116, < 0·001	1668·5	447·9	1685·9	480·3	–17·4	474·6	116, < 0·001	0·15
Fat (g)	44·5	20·6	46·4	25·4	–1·9	25·5	68, < 0·001	42·9	23·1	39·0	20·4	3·9	25·6	49, < 0·001	0·08
Protein (g)	72·6	32·2	76·3	29·6	–3·7	34·8	92, < 0·001	75·3	37·0	67·4	22·6	7·9	40·6	91, < 0·001	0·01
Carbohydrates (g)	258·7	82·8	277·1	100·2	–18·4	70·1	115, < 0·001	242·6	82·4	261·3	95·4	–18·7	75·3	110, < 0·001	0·65
Total fibre (g)	7·0	4·8	7·7	7·1	–0·7	5·6	45, < 0·001	7·4	4·4	7·6	6·6	–0·3	4·9	46, 0·017	0·19
Vitamin A (mcg RAE)	408·5	302·2	416·1	415·3	–7·6	314·4	84, 0·18	456·0	342·4	394·2	221·8	61·9	289·3	80, 0·79	N/A
Vitamin C (mg)	91·2	68·8	100·1	86·8	–8·9	67·9	68, < 0·001	85·0	60·1	95·5	88·6	–10·5	63·8	73, < 0·001	0·41
Ca (mg)	468·8	205·0	528·0	233·2	–59·2	220·3	106, < 0·001	476·1	195·2	484·6	179·6	–8·5	207·7	103, < 0·001	0·09
Fe (mg)	12·7	4·4	13·9	5·9	–1·3	5·1	70, < 0·001	13·4	5·6	12·5	4·2	0·9	4·9	61, < 0·001	0·01
Zn (mg)	9·2	3·4	10·2	4·5	–1	3·7	57, < 0·001	8·8	2·6	9·2	3·4	–0·4	3·1	63, < 0·001	0·19

PAPI, pen-and-paper interview; INDDEX, International Dietary Data Expansion; WFR, weighed food record; RAE, retinol activity equivalent.

a Note the WFR is the minuend, and therefore a negative difference between WFR and 24HR modalities (PAPI and INDDEX24) indicates an overestimate, while a positive number indicates an underestimate.

b Equivalence test *P*-value is from the paired two one-sided t test (TOST) reported using the natural log geometric mean and 10% bound.

c The difference-in-difference *P*-value is calculated based on the first difference between each respective 24HR modality (PAPI and INDDEX24) and the WFR and the second difference based on those differences ((WFR-PAPI) – (WFR-INDDEX24)).

The difference-in-difference regression results, also presented in Table 2, showed that none of the relative differences between the two arms ((WFR-PAPI) – (WFR-INDDEX24)) was statistically significant, with the exception of protein and Fe. In the case of protein, the PAPI diverged less from the WFR than INDDEX24 (–3·7 *v*. 7·9 g), whereas for Fe INDDEX24 diverged significantly less than PAPI from the WFR (0·9 *v*. −1·3 mg). Overall, the differences between each 24HR modality (INDDEX24 and PAPI) compared with the WFR, and those differences compared with each other, were small.

The small differences are visible in a series of Bland–Altman plots (online Supplementary Fig. A–J) which show individual differences between the two methods and were used to examine the mean bias, limits of agreement and the distribution of bias. Side-by-side comparisons for both WFR-PAPI and WFR-INDDEX24 showed similar patterns. The plots for protein and fat indicated a greater bias with increased intake. This bias, although small, was inverted, with higher intakes for the WFR method on the PAPI side and higher intakes for the recall method on the INDDEX24 side. Carbohydrate, on the other hand, exhibited a small mean difference below zero for both methods, indicating a pattern of higher intakes for the WFR method. Consistent bias towards higher WFR values for protein, fat and carbohydrates are illustrated in the energy plot for the PAPI-WFR comparison. For micronutrients, 95 % of values remained within the limits of agreement and the mean difference in relation to zero (bias) remained small.

On an individual level, 26 % of PAPI respondents and 32 % of INDDEX24 respondents had energy intakes within 10 % of their WFR recalls, while a total of 53 % of PAPI and 59 % of INDDEX24 respondents reported energy intakes within 20 % of the benchmark (Table 3). For the nutrient estimates, the percentage of respondents within 10 % of their WFR varied by modality. For fat, fibre, vitamin A and Fe, the PAPI tended to have slightly more respondents within 10 % of their WFR, while for carbohydrates and Ca more respondents in the INDDEX24 arm were within 10 % of their WFR values. For the remaining nutrients (protein, vitamin C and Zn), item count and gram amount, the percentage of respondents within 10 % of their WFR was essentially the same for PAPI and INDDEX24. While overall, the group averages in Table 2 indicated slight overestimates for many nutrients across both PAPI and INDDEX24, the individual level error results in Table 3 – when disaggregated by overestimates *v*. underestimates – show that at an individual level there were non-trivial underestimates, particularly in the outer tails of the distribution (comparing < –50 % error to > +50 %). This highlights that as error estimates grow larger, both the PAPI and INDDEX24 24HR modalities were more likely to underestimate, rather than overestimate, compared with the WFR (online Supporting Material, Table S1).

**Table 3.** Percent of respondents falling within ranges of percent error in estimating energy and nutrient intakes with PAPI and INDDEX24 24HR modalities compared with WFR (*n* 234) in Thanh Oai, Viet Nam

	Arm 1: PAPI (*n* 117)						Arm 2: INDDEX24 (*n* 117)					
	0–±10%	±10·1–20 %	±20·1–30 %	±30·1–40 %	±40·1–50 %	>±50 %	0–±10 %	±10·1–20 %	±20·1–30 %	±30·1–40 %	±40·1–50 %	>±50 %
Item count	41·0	30·8	14·5	11·1	1·7	0·9	41·0	27·4	13·7	11·1	4·3	2·6
Gram amount	39·3	26·5	16·2	6·0	5·1	6·8	36·8	25·7	16·2	11·1	3·4	6·8
Energy (kcals)	25·6	27·4	18·8	11·1	8·6	8·6	31·6	27·4	18·0	7·7	8·5	6·8
Fat (g)	20·5	18·8	11·1	12·8	10·3	26·5	11·1	12·0	12·0	16·2	12·8	35·9
Protein (g)	24·8	24·8	18·8	11·1	5·1	15·4	23·9	20·5	17·1	13·7	8·5	16·2
Carbohydrates (g)	27·4	29·9	15·4	13·7	7·7	6·0	39·3	29·9	8·5	9·4	1·7	11·1
Total fibre (g)	28·2	17·1	12·0	12·8	11·1	18·8	23·9	18·8	15·4	13·7	9·4	18·8
Vitamin A (mcg RAE)	23·9	12·0	7·7	18·8	8·5	29·1	12·8	14·5	17·1	11·1	14·5	29·9
Vitamin C (mg)	17·1	19·7	10·3	16·2	8·5	28·2	18·0	16·2	12·0	12·0	11·1	30·8
Ca (mg)	24·8	29·1	10·3	7·7	10·3	17·9	27·4	23·9	17·1	10·3	6·0	15·4
Fe (mg)	27·4	20·5	21·4	6·0	6·8	17·9	20·5	24·8	16·2	17·1	8·6	12·8
Zn (mg)	26·5	22·2	18·0	7·7	12·8	12·8	27·4	25·6	13·7	11·1	6·8	15·4

PAPI, pen-and-paper interview; INDDEX, International Dietary Data Expansion; WFR, weighed food record; RAE, retinol activity equivalent.

### Differences in estimates of food group intakes by pen-and-paper interview *v.* INDDEX24 24-h dietary recall modalities compared with weighed food record

The median percent of energy intake from the major FAO/WHO GIFT food groups provides further information about differences between the 24HR modalities (Table 4). These results again underscore the negligible absolute and relative average differences between the two 24HR modalities and the WFR: the absolute differences in energy intake between the PAPI-WFR and INDDEX24-WFR did not exceed +/–3 percentage points for any food group. Neither PAPI nor INDDEX24 24HR modality was statistically different from WFR for the majority of the food groups.

**Table 4.** Median percent of energy intake from major FAO/WHO GIFT food groups (*n* 234) in Thanh Oai, Viet Nam (Median values and percentiles)

	Arm 1: PAPI (*n* 117)							Arm 2: INDDEX24 (*n* 117)						
Food Group^a^	Respondent consuming (%)	WFR Median^b^	WFR 25th, 75th	PAPI Median	PAPI 25th, 75th	Median difference^c^	WRS test *P*-value^d^	Respondent consuming (%)	WFR Median	WFR 25th, 75th	INDDEX24 Median	INDDEX24 25th, 75th	Median difference	WRS test *P*-value
Cereals	100	54·8	47·5, 62·8	56·8	43·6, 62·8	–2·0	0·96	100	55·6	39·7, 64·6	56·8	46·1, 67·5	–1·2	0·02
Vegetables	100	3·7	2·5, 5·7	3·1	2·2, 5·0	0·6	0·06	100	4·0	2·6, 6·3	3·7	2·3, 5·5	0·3	0·19
Beverages	100	0·3	0·1, 0·8	0·6	0·3, 1·2	–0·3	0·001	100	0·2	0·1, 0·8	0·8	0·2, 1·3	–0·6	< 0·001
Spices and condiments	100	0·2	0·1, 0·5	0·3	0·1, 0·6	–0·1	0·02	100	0·2	0·1, 0·6	0·4	0·1, 0·8	–0·2	0·26
Meat	99·1	13·2	7·8, 20·4	13·7	6·8, 21·3	–0·5	0·71	95·7	14·5	8·3, 22·3	11·8	7·3, 21·7	2·7	0·19
Fats and oils	94·9	5·6	3·3, 10·1	4·7	2·9, 8·4	0·9	0·02	90·6	5·3	2·8, 9·0	3·9	2·0, 7·9	1·4	0·11
Fruits	70·9	5·3	3·0, 7·6	5·7	3·4, 9·3	–0·4	0·19	70·9	4·9	2·1, 8·5	5·6	2·3, 9·4	–0·7	0·91
Pulses and seeds	63·2	7·1	3·6, 10·9	6·6	3·7, 10·4	0·5	0·42	60·7	7·3	3·7, 12·8	5·8	3·2, 12·0	1·5	0·03
Eggs and egg products	55·6	3·5	2·1, 5·3	3·0	2·0, 5·2	0·5	0·34	54·7	3·5	1·8, 5·3	3·0	1·7, 5·1	0·5	0·29
Sweets & sugars	47·0	0·7	0·1, 5·0	0·7	0·2, 3·7	0·0	0·77	53·0	0·5	0·0, 2·4	1·0	0·2, 3·7	–0·5	0·40
Fish and shellfish	27·4	2·3	0·5, 3·5	2·7	1·4, 8·1	–0·4	0·39	34·2	3·4	1·2, 7·6	2·5	1·2, 6·3	0·9	0·49
Milk and milk products	27·4	4·3	2·3, 9·4	4·1	3·2, 9·3	0·2	0·61	17·1	5·2	3·8, 10·8	7·2	4·2, 8·7	–2·0	0·57
Roots and tubers	17·9	5·2	2·3, 6·1	3·8	1·6, 5·1	1·4	0·15	16·2	5·3	1·0, 6·8	3·9	1·9, 9·3	1·4	0·82

PAPI, pen-and-paper interview; INDDEX, International Dietary Data Expansion; WFR, weighed food record; WRS, Wilcoxon’s rank sum test.

a Results for three food groups are not shown here as they were consumed by less than 5 % of respondents in each arm: savoury snacks (1·7 % PAPI and 3·4 % INDDEX24), insects and grubs (0·9 % PAPI and 0·9 % INDDEX24), and food additives (0·9 % PAPI and 0·0 % INDDEX24).

b Median results are based on all respondents in that arm (consumers and non-consumers).

c Note the WFR is the minuend, and therefore a negative difference between WFR and 24HR modalities indicates an overestimate and a positive number indicates and underestimate.

d WRS = Wilcoxon Rank Sum Test is a nonparametric test that compares two paired groups.

### Cost-effectiveness of pen-and-paper interview *v.* INDDEX24

As detailed in Adams *et al.*, 2021^(51)^, the total economic cost of using INDDEX24 to conduct the 24HR was $111 004 (2020 USD; *n* 147), compared with $120 483 (2020 USD; *n* 147) for PAPI. While the non-time costs (e.g. equipment) were higher for INDDEX24 than PAPI, INDDEX24 had lower personnel costs, leading to a $9479 cost savings to conduct the 24HR using INDDEX24 compared with PAPI. The average percent accuracy in estimating the gram amount and the ten-nutrient composite measure of nutrient intake was slightly higher using the INDDEX24 modality than the PAPI modality, while the average percent accuracy in estimating item count was slightly higher using PAPI (Table 5). However, given the cost savings associated with INDDEX24, INDDEX24 was more cost-effective than PAPI for all three outcomes.

**Table 5.** Cost-effectiveness of conducting a 24HR using PAPI and INDDEX24 in Viet Nam

Outcome	Modality	Average percent accuracy	Cost-effectiveness^*^ (2019 USD)
Gram amount	INDDEX24	97	1143
	PAPI	96	1261
	Difference^†^	2	–118
Item count	INDDEX24	91	1220
	PAPI	92	1313
	Difference	–1	–93
Ten-nutrient composite measure of nutrient intake^‡^	INDDEX24	93	1194
	PAPI	92	1306
	Difference	1	–113

24HR, 24-h dietary recalls; PAPI, pen-and-paper interview; INDDEX, International Dietary Data Expansion.

* See Adams *et al.* ^(51)^ for detailed cost information. Cost-effectiveness was calculated as cost per average percentage point of accuracy.

† The difference was calculated as INDDEX24 minus PAPI. All estimates are rounded to the nearest whole number.

‡ Ten-nutrient composite measure of nutrient intake is calculated as the overall average of the average percent accuracy of PAPI (or of INDDEX24) in estimating intake for energy, fat, protein, carbohydrate, fibre, vitamin A, vitamin C, Ca, Fe and Zn relative to intake based on the weighed food record.

On a nutrient-specific basis (online Supporting Material, Table S2), there was variability in the average percent accuracy achieved using INDDEX24 relative to PAPI (INDDEX24 had a higher average percent accuracy for energy, fibre, Ca, Fe and Zn, while PAPI had a higher average percent accuracy for fat, protein, carbohydrate, vitamin A and vitamin C). However, INDDEX24 was more cost-effective than PAPI for all nutrients except vitamin A.

## Discussion

To our knowledge, this study was the first to assess whether using a technology-enabled approach to dietary assessment in an LMIC context can confer added benefits to a PAPI approach in accuracy and cost-effectiveness.

The results showed that the INDDEX24 technology-enabled approach was an accurate modality for assessing food and nutrient intake at a population level. While the accuracy of INDDEX24 and PAPI were similar, the INDDEX24 Dietary Assessment Platform cost less, which meant that INDDEX24 was the more cost-effective modality for collecting and processing accurate dietary recall data.

Though INDDEX24 performed better than the PAPI 24HR across a number of different measures of accuracy, the two modalities were not significantly different from one another on most measures. Our individual-level analyses showed non-systematic overestimation and underestimation for both INDDEX24 and PAPI for most foods and nutrients which, on average, resulted in estimates that did not deviate significantly from the benchmark results. The results of other 24HR validation studies in LMIC show similar variability in under- *v*. over-reporting as our study. Some have documented systematic under-reporting^(56–62)^, while others found that the 24HR resulted in non-significant differences, on average, from a WFR benchmark, including some negligible over-reporting at a population level^(63–68)^. An extreme value for vitamin A intake in INDDEX24 but not in PAPI contributed to the poorer performance of INDDEX24 for this nutrient. There are a number of plausible explanations for extreme values in one or the other modality, including the possibility of an omission or intrusion of an easily overlooked or rarely consumed food high in vitamin A by an INDDEX24 respondent but not a PAPI respondent. Furthermore, this study did not conduct multiple 24HR on the same individuals because the aim was not to calculate usual intake but rather gauge the relative accuracy of a single-day recall by modality relative to the WFR. Conducting multiple non-sequential days of recall to enable the estimation of usual intake typically helps smooth intra-individual variability and would be recommended for most dietary surveys that use either INDDEX24 or PAPI modalities.

There are several reasons why the PAPI and INDDEX24 might have performed similarly in terms of accuracy. First, both modalities were carefully designed to collect 24HR data using the same rigorous multiple-pass method. A more typical PAPI is of lower technical quality and may have diverged more dramatically from both the INDDEX24 results and the WFR. Second, in our study, the same enumerators received intensive, high-quality training on both modalities and alternately carried out both PAPI and INDDEX24 interviews. This cross-training likely benefited both modalities. The alternative, randomising interviewers to implement either INDDEX24 or PAPI (but not both), was rejected due to the concern for systematic bias stemming from enumerator skill levels and interview execution. Third, both modalities likely benefited from the systematic approach to developing dietary reference data that is required by the FMDB component of INDDEX24. Fourth, some of the convergence in results may be due to the use of standard recipes for both INDDEX24 and PAPI, despite the fact that standard recipes are not typical of most PAPI approaches.

While no other documented studies to date have compared a technology-enabled approach with a PAPI 24HR and against a benchmark method in an LMIC context, a few other studies have compared data collection modalities or a CAPI approach on its own to a benchmark method such as WFR or biomarkers^(18,69–71)^. The vast majority of these studies were conducted in high-income countries, and none assessed multiple modalities with a benchmark in a single evaluative framework. Of the small number of studies comparing a CAPI and PAPI 24HR data collection modality in an LMIC without a benchmark, Zang *et al.* (2015)^(18)^ did not attempt to assess accuracy but found that the CAPI and PAPI produced similar results relating to acceptability and feasibility of use among adults in the Shanghai, China region. Htet *et al.* (2019)^(19)^ found that applying a CAPI multiple-pass 24HR method in Indonesia to assess children’s diets resulted in a higher percentage of children classified as acceptable energy reporters (within 95 % CI of modelled total energy expenditure) when using CAPI *v*. PAPI. As in our study, they found no significant differences between estimated mean energy and nutrient intakes for the technology-enabled CAPI *v*. PAPI.

There are some potential limitations to the study results, in addition to the potential influence of instruments and experience across enumerators implementing INDDEX24 and PAPI described above. The INDDEX24 sample was slightly more educated than the PAPI sample, albeit non-significantly so. This difference in education level might have affected the ability of INDDEX24 respondents to better recall foods and nutrients relative to PAPI respondents. At the respondent level, there was a risk of ‘priming bias’, where the WFR conducted the previous day may have prompted respondents to better remember what they ate in the recall. While this explanation seems intuitively plausible, a 1994 study by Ferguson in Malawi that tested for priming bias from an observer-weighed food record found no evidence that this was a concern^(72)^. Priming bias would have been faced similarly across both the INDDEX24 and PAPI arms and, therefore, is not a threat to the overall objective of the study, which was to assess relative accuracy of the two modalities. And, while ‘priming’ is a factor to be explored further in future studies that do seek to assess the accuracy of 24HR methods, there is evidence to suggest that intentional priming (e.g. asking respondents to photograph the foods eaten, maintain a detailed food diary (for literate populations) or place a ‘check’ beside illustrations of foods the 1–3 d prior to the 24HR) could be a useful means of improving the accuracy of respondents’ recall, a desirable outcome in itself^(73,74)^.

Similiarly, other potential limitations, including reactivity bias (where WFR respondents may have modified their consumption while under direct observation by an enumerator) and the single rather than repeat WFR-24HR visits required to construct indicators of ‘usual intake’, were not an issue for the primary objective of our study, which was to test the relative difference between INDDEX24 and PAPI modalities. These factors could affect the absolute results of the 24HR compared with WFR benchmark, in terms of the accuracy of average intake estimates overall. A stronger benchmark than the WFR, that avoids some types of WFR-specific error as well as shared error with the 24HR modalities, would have been a biomarker method such as doubly labelled water or urinary nitrogen. However, these biomarker methods were ruled out for the current study, primarily because they would not have allowed us to disentangle the sources of error in the test modalities, which the WFR was better suited to enable.

Viet Nam is a lower-middle-income country with a complex food system, an intricate diet and customary eating practices (e.g. eating food from a common bowl with chopsticks) that create numerous challenges for accurate dietary assessment, whether done through direct observation and weighing or by recall. That the INDDEX24 Dietary Assessment Platform performed well in this context, together with similar results from a parallel study conducted by many of the same researchers in Burkina Faso^(20)^, lends confidence to its use in other LMIC with potentially less varied eating patterns and overall diets. Additional testing and validation of INDDEX24 is warranted, on surveys of different scales (e.g. nationally representative studies) and for other population groups (e.g. men and children). Replication of the validation protocol in a heavily urban population where packaged, processed and street foods form a large part of the diet will also offer useful insights to improve INDDEX24. It would be helpful to perform additional validation in contexts where respondents speak multiple languages within or across study sites. The use of a common language for interviewing in Viet Nam (Vietnamese) meant the relative effects of language switching in the INDDEX24 Mobile App on accuracy and cost were not tested. INDDEX24 offers enumerators the flexibility to toggle among up to four different languages within a single app, a feature that could confer added benefit to the process over a PAPI that would need multiple paper questionnaires in a range of languages. This study compared INDDEX24 with PAPI, since PAPI is still the most widely used method in LMIC. Further research could assess the relative accuracy of INDDEX24 compared with other technology-enabled approaches for dietary assessment including other ‘innovative’ technologies such as active or passive food imaging that do not rely on recall.

Our cost and cost-effectiveness analyses suggested that the use of INDDEX24 cost $9000 less per respondent than the PAPI when the dietary reference data were largely assembled from scratch. Our modelling projections suggest that the total cost for INDDEX24 would be approximately $40 000 less than the PAPI approach when 75 % of the needed dietary reference data were available in the FMDB as a starting point (see Adams *et al.*, 2021 for details of this and other modelled cost scenarios). Populating the FMDB with country-specific data is a priority since, as dietary reference data are pre-populated in the FMDB, the absolute cost of using INDDEX24 will likely decline significantly, further improving the cost-effectiveness of INDDEX24 relative to PAPI. Furthermore, the detailed comparative cost models of INDDEX24 and PAPI constructed by Adams *et al.*, 2021 suggest that economies of scale favour INDDEX24; in larger studies, the cost of implementing a 24HR declines at a greater rate for INDDEX24 than for PAPI, further increasing the overall cost-effectiveness of INDDEX24.

### Conclusion

The INDDEX24 Dietary Assessment Platform was developed with the aim of streamlining and standardising the collection of 24HR data, to enable more LMIC researchers and governments to collect quantitative dietary data to inform a wide range of health, nutrition, agricultural, food safety and environmental policies. This validation study in Viet Nam has shown that the INDDEX24 Dietary Assessment Platform yields results that are as accurate as a standard PAPI approach while costing less. The evidence from this validation study offers positive reinforcement of the benefit of adopting INDDEX24 for implementing high-quality, quantitative dietary assessments.

## Supplementary material

For supplementary material accompanying this paper visit https://doi.org/10.1017/S0007114522001507. click here to view supplementary material

## References

[1] Popkin B , Adair L & Ng S (2012) Global nutrition transition and the pandemic of obesity in developing countries. Nutr Rev 70, 3–21. 22221213 10.1111/j.1753-4887.2011.00456.x PMC3257829

[2] Di Cesare M (2019) Global trends of chronic non-communicable diseases risk factors. Eur J Public Health 29, S185–S196.

[3] Micha R , Mannar V , Afshin A , et al. (2020) Global Nutrition Report: Action on Equity to End Malnutrition. https://globalnutritionreport.org/reports/2020-global-nutrition-report/ (accessed March 2021).

[4] Coates JC , Colaiezzi BA , Bell W , et al. (2017) Overcoming dietary assessment challenges in low-income countries: technological solutions proposed by the International Dietary Data Expansion (INDDEX) Project. Nutrients 9, 289–304. 28300759 10.3390/nu9030289 PMC5372952

[5] HLPE (2017) Nutrition and Food Systems. A Report by the High Level Panel of Experts on Food Security and Nutrition of the Committee on World Food Security, Rome. http://www.fao.org/3/i7846e/i7846e.pdf (accessed March 2021).

[6] FAO/WHO (2022) FAO/WHO GIFT Individual Quantitative Food Consumption Data Inventory. http://www.fao.org/gift-individual-food-consumption/inventory-of-surveys/en/ (accessed March 2022).

[7] Bradley J , Simpson E , Poliakov I , et al. (2016) Comparison of INTAKE24 (an Online 24-h Dietary Recall Tool) with interviewer-led 24-h recall in 11–24 year-old. Nutrients 8, 358–371. 27294952 10.3390/nu8060358 PMC4924199

[8] Carrard I , Farina E , Danuser B , et al. (2017) Development and evaluation of e-CA, an electronic mobile-based food record. Nutrients 9, 76. 28106767 10.3390/nu9010076 PMC5295120

[9] Carter MC , Albar SA , Morris MA , et al. (2015) Development of a UK online 24-h dietary assessment tool: myfood24. Nutrients 7, 4016–4032. 26024292 10.3390/nu7064016 PMC4488770

[10] Savard C , Lemieux S , Lafrenière J , et al. (2018) Validation of a self-administered web-based 24-hour dietary recall among pregnant women. BMC Pregnancy Childbirth 18, 112. 29685127 10.1186/s12884-018-1741-1 PMC5913813

[11] Slimani N , Casagrande C , Nicolas G , et al. (2011) The standardized computerized 24-h dietary recall method EPIC-Soft adapted for pan-European dietary monitoring. Eur J Clin Nutr 65, S5. 21731006 10.1038/ejcn.2011.83

[12] Amoutzopoulos B , Steer T , Roberts C , et al. (2018) Traditional methods *v.* new technologies – dilemmas for dietary assessment in large-scale nutrition surveys and studies: a report following an international panel discussion at the 9th International Conference on Diet and Activity Methods (ICDAM9), Brisbane, 3 September 2015. J Nutr Sci 7, e11. 29686860 10.1017/jns.2018.4 PMC5906559

[13] Foster E , Lee C , Imamura F , et al. (2019) Validity and reliability of an online self-report 24-h dietary recall method (Intake24): a doubly labelled water study and repeated-measures analysis. J Nutr Sci 8, e29. 31501691 10.1017/jns.2019.20 PMC6722486

[14] Del Gobbo LC , Khatibzadeh S , Imamura F , et al. (2015) Assessing global dietary habits: a comparison of national estimates from the FAO and the Global Dietary Database. Am J Clin Nutr 101, 1038–1046. 25788002 10.3945/ajcn.114.087403 PMC4409685

[15] Micha R , Coates J , Leclercq C , et al. (2018) Global dietary surveillance: data gaps and challenges. Food Nutr Bull 39, 175–205. 29478333 10.1177/0379572117752986

[16] Caswell BL , Talegawkar SA , Dyer B , et al. (2015) Assessing child nutrient intakes using a tablet-based 24-hour recall tool in Rural Zambia. Food Nutr Bull 36, 467–480. 26487637 10.1177/0379572115612631

[17] Harris-Fry H , Beard B , Harrisson T , et al. (2018) Smartphone tool to collect repeated 24 h dietary recall data in Nepal. Public Health Nutr 21, 260–272. 28854993 10.1017/S136898001700204X PMC10261075

[18] Zang J , Song J , Wang Z , et al. (2015) Acceptability and feasibility of smartphone-assisted 24 h recalls in the Chinese population. Public Health Nutr 18, 3272–3279. 25857612 10.1017/S1368980015000907 PMC4600407

[19] Htet MK , Fahmida U , Do T , et al. (2019) The use of tablet-based multiple-pass 24-hour dietary recall application (MP24diet) to collect dietary intake of children under two years old in the prospective cohort study in Indonesia. Nutrients 11, 2889. 31783608 10.3390/nu11122889 PMC6950229

[20] Rogers B , Somé J , Bakun P , et al. (2021) Validation of the INDDEX24 mobile app *v.* a pen-and-paper 24-hour dietary recall using the weighed food record as a benchmark in Burkina Faso. Br J Nutr 1–15. doi: 10.1017/S0007114521004700. PMC9592947 34823617

[21] World Bank & Map Vietnam (2022) mapVIETNAM – World Bank Group. http://www.worldbank.org/mapvietnam/ (accessed February, 2022).

[22] Gibson RS (2005) Principles of Nutritional Assessment, 2nd ed. New York: Oxford University Press.

[23] Wrieden W , Peace H , Armstrong J , et al. (2003) A Short Review of Dietary Assessment Methods used in National and Scottish Research Studies. https://www.food.gov.uk/sites/default/files/multimedia/pdfs/scotdietassessmethods.pdf (accessed March 2021).

[24] Gibson RS & Ferguson EL (2008) An Interactive 24-Hour Recall for Assessing the Adequacy of Iron and Zinc Intakes in Developing Countries. http://www.ifpri.org/sites/default/files/publications/tech08.pdf (accessed March 2021).

[25] Gibson RS , Charrondiere R & Bell W (2017) Measurement errors in dietary assessment using self-reported 24-hour recalls in low-income countries and strategies for their prevention. Adv Nutr 8, 11. 10.3945/an.117.016980 PMC5683000 29141979

[26] Wafa S , Bell W , Coates JC , et al. (2018) Cognitive processes of respondents in tablet-based 24-hour dietary recalls in Burkina Faso and Viet Nam. Curr Dev Nutr 5, 620.

[27] INDDEX (2022) Global Food Matters Database. https://globalfoodmattersdatabase.org/ws/91/foods/# (accessed March 2022).

[28] National Institute of Nutrition & Ministry of Health (2017) Vietnamese Food Composition Table. Hanoi: Medical Publishing House.

[29] National Institute of Nutrition & Ministry of Health (2014) Food Photo Atlas for Dietary Assessment of Children 2–5 Years Old. Hanoi: Medical Publishing House.

[30] National Institute of Nutrition & Ministry of Health (2016) Nutritive Value of 500 Common Dishes. Hanoi: Medical Publishing House.

[31] National Institute of Nutrition & Ministry of Health (2017) Yield Factor. Hanoi: Medical Publishing House.

[32] Ho Chi Minh City’s Nutrition Center (2017) Nutritive Value of Common Street Foods. Ho Chi Minh City: Medical Publishing House.

[33] FAO (2015) FAO/INFOODS/TGI Global Supplement Database – Version 1. Rome: FAO.

[34] FAO (2016) FAO/INFOODS Global Food Composition Database for Fish and Shellfish Version 1.0 – uFiSh1.0. Rome: FAO.

[35] FAO (2017) FAO/INFOODS Global Food Composition Database for Pulses Version 1.0 – uPulses 1.0. Rome: FAO.

[36] Haytowitz DB , Ahuja JKC , Wu X , et al. (2019) *USDA National Nutrient Database for Standard Reference, Legacy Release. Nutrient Data Laboratory*. Beltsville, MD: Human Nutrition Research Center, ARS, USDA; available at https://data.nal.usda.gov/dataset/usda-national-nutrient-database-standard-reference-legacy-release (accessed July 2019).

[37] Institute of Nutrition, Mahidol University (2014) ASEAN Food Composition Database, Electronic Version 1, February 2014, Thailand. http://www.inmu.mahidol.ac.th/aseanfoods/doc/ASEAN_FCD_V1_2014.pdf (accessed July 2019).

[38] Judprasong K , Puwastien P , Rojroongwasinkul N , et al. (2015) Thai Food Composition Database, Institute of Nutrition, Mahidol University, Online Version 2. http://www.inmu.mahidol.ac.th/thaifcd (accessed July 2019).

[39] Rural Development Administration & Rural Resources Development Institute (2011) Korean Standard Food Composition Table, Suwon, 8th ed. http://koreanfood.rda.go.kr/eng/fctFoodSrchEng/engMain (accessed July 2019).

[40] Ministry of Education, Culture, Sports, Science and Technology (2015) Standard Tables of Food Composition in Japan (7th Revised Edition), Documentation and Table. https://www.mext.go.jp/en/policy/science_technology/policy/title01/detail01/sdetail01/sdetail01/1385122.htm (accessed July 2019).

[41] FAO (2012) FAO/INFOODS Guidelines for Food Matching, Version 1.2. Rome, Italy. http://www.fao.org/docrep/017/ap805e/ap805e.pdf (accessed July 2019).

[42] U.S. Census Bureau (2021) Census and Survey Processing System (CSPro). https://www.census.gov/data/software/cspro.html (accessed September 2021).

[43] Bell W , Coates JC , Rogers BL , et al. (2019) Getting the food list ‘right’: an approach for the development of nutrition-relevant food lists for household consumption and expenditure surveys. Public Health Nutr 22, 246–256. 30394251 10.1017/S1368980018002847 PMC6390398

[44] Bermudez OI , Lividini K , Smitz MF , et al. (2012) Estimating micronutrient intakes from Household Consumption and Expenditures Surveys (HCES): an example from Bangladesh. Food Nutr Bull 33, S208–S221. 23193772 10.1177/15648265120333S209

[45] Tucker KL , Bianchi LA , Maras J , et al. (1998) Adaptation of a food frequency questionnaire to assess diets of Puerto Rican and non-Hispanic adults. Am J Epidemiol 148, 507–518. 9737563 10.1093/oxfordjournals.aje.a009676

[46] Bland JM & Altman DG (1999) Measuring agreement in method comparison studies. Stat Meth Med Res 8, 135–160. 10.1177/096228029900800204 10501650

[47] Bland JM & Altman DG (2007) Agreement between methods of measurement with multiple observations per individual. J Biopharm Stat 17, 571–582. 17613642 10.1080/10543400701329422

[48] Arsenault JE , Moursi M , Olney DK , et al. (2020) Validation of 24-h dietary recall for estimating nutrient intakes and adequacy in adolescents in Burkina Faso. Matern Child Nutr 16, e13014. 32337835 10.1111/mcn.13014 PMC7503205

[49] Batterham MJ , Van Loo C , Charlton KE , et al. (2016) Improved interpretation of studies comparing methods of dietary assessment: combining equivalence testing with the limits of agreement. Br J Nutr 115, 1273–1280. 26879342 10.1017/S0007114516000040

[50] Tugault-Lafleur CN , Black JL & Barr SI (2017) A systematic review of methods to assess children’s diets in the school context. Adv Nutr 8, 63–79. 28096128 10.3945/an.116.013144 PMC5227974

[51] Adams K , Bell W , Somé J , et al. (2021) The cost of conducting a 24-hour dietary recall using INDDEX24, a mobile dietary assessment platform, compared to pen-and-paper in Viet Nam and Burkina Faso. Curr Dev Nutr 5, S620. 10.1017/S0007114522001362 PMC9876804 35508922

[52] World Health Organization (2003) *Making Choices in Health: WHO Guide to Cost-Effectiveness Analysis*. Geneva: WHO; available at https://apps.who.int/iris/handle/10665/42699 (accessed June 2020).

[53] Hatløy A , Torheim LE & Oshaug A (1998) Food variety – a good indicator of nutritional adequacy of the diet? A case study from an urban area in Mali, West Africa. Eur J Clin Nutr 52, 891–898. 9881884 10.1038/sj.ejcn.1600662

[54] Steyn NP , Nel J , Labadarios D , et al. (2014) Which dietary diversity indicator is best to assess micronutrient adequacy in children 1–9 years? Nutrition 30, 55–60. 24290599 10.1016/j.nut.2013.06.002

[55] Mirmiran P Azadbakht L , Esmaillzadeh A , et al. (2004) Dietary diversity score in adolescents – a good indicator of the nutritional adequacy of diets: Tehran lipid and glucose study. Asia Pac J Clin Nutr 13, 56–60. 15003915

[56] Alemayehu AA , Abebe Y & Gibson RS (2011) A 24-h recall does not provide a valid estimate of absolute nutrient intakes for rural women in southern Ethiopia. Nutrition 27, 919–924. 21295444 10.1016/j.nut.2010.10.015

[57] Carvalho MA , Baranowski T , Foster E , et al. (2014) Validation of the Portuguese self-administered computerised 24-hour dietary recall among second-, third- and fourth-grade children. J Hum Nutr Diet 28, 666–74. 25420921 10.1111/jhn.12280

[58] Gewa CA , Murphy SP & Neumann CG (2009) A comparison of weighed and recalled intakes for schoolchildren and mothers in rural Kenya. Public Health Nutr 12, 1197–1204. 18789170 10.1017/S1368980008003698

[59] Jayawardena R (2016) Comparison dietary assessment methods in Sri Lankan adults: use of 24-hour dietary recall and 7-day weighed intake. BMC Nutr 2, 1–4.

[60] Orcholski L , Luke A , Plange-Rhule J , et al. (2015) Under-reporting of dietary energy intake in five populations of the African diaspora. Br J Nutr 113, 464–472. 25585294 10.1017/S000711451400405X PMC4670081

[61] Pfrimer K , Vilela M , Resende CM , et al. (2015) Under-reporting of food intake and body fatness in independent older people: a doubly labelled water study. Age Ageing 44, 103–108. 25341675 10.1093/ageing/afu142

[62] Scagliusi FB , Ferriolli E , Pfrimer K , et al. (2008) Underreporting of energy intake in Brazilian women varies according to dietary assessment: a cross-sectional study using doubly labeled water. J Am Diet Assoc 108, 2031–2040. 19027406 10.1016/j.jada.2008.09.012

[63] Dop M-C , Milan CH , Milan CL , et al. (1994) The 24-hour recall for Senegalese weanlings: a validation exercise. Eur J Clin Nutr 48, 643–653. 8001521

[64] Ferguson EL , Gibson RS , Ounpuu S , et al. (1989) The validity of the 24 hour recall for estimating the energy and selected nutrient intakes of a group of rural Malawian preschool children. Ecol Food Nutr 23, 273–285.

[65] Ferguson EL , Gadowsky SL , Huddle JM , et al. (1995) An interactive 24-h recall technique for assessing the adequacy of trace mineral intakes of rural Malawian women; its advantages and limitations. Eur J Clin Nutr 49, 565–578. 7588507

[66] Hemsworth J , Arimond M , Kumwenda C , et al. (2018) Comparison of an interactive 24-h recall and weighed food record for measuring energy and nutrient intakes from complementary foods among 9–10-month-old Malawian infants consuming lipid-based nutrient supplements. Br J Nutr 120, 1262–1271. 30350761 10.1017/S0007114518002374

[67] Nightingale H , Walsh KJ , Olupot-Olupot P , et al. (2016) Validation of triple pass 24-hour dietary recall in Ugandan children by simultaneous weighed food assessment. BMC Nutr 2, 1–9. 10.1186/s40795-016-0092-4 PMC5081093 27795836

[68] Thakwalakwa CM , Kuusipalo HM , Maleta KM , et al. (2012) The validity of a structured interactive 24-hour recall in estimating energy and nutrient intakes in 15-month-old rural Malawian children. Matern Child Nutr 8, 380–389. 21323866 10.1111/j.1740-8709.2010.00283.x PMC6860657

[69] Kirkpatrick SI , Subar AF , Douglass D , et al. (2014) Performance of the automated self-administered 24-hour recall relative to a measure of true intakes and to an interviewer-administered 24-h recall. Am J Clin Nutr 100, 233–240. 24787491 10.3945/ajcn.114.083238 PMC4144101

[70] Moshfegh AJ , Rhodes DG , Baer DJ , et al. (2008) The US Department of Agriculture automated multiple-pass method reduces bias in the collection of energy intakes. Am J Clin Nutr 88, 324–32. 18689367 10.1093/ajcn/88.2.324

[71] Timon CM , van den Barg R , Blain RJ , et al. (2016) A review of the design and validation of web- and computer-based 24-h dietary recall tools. 29, 268–280. 10.1017/S0954422416000172 27955721

[72] Ferguson EL , Gibson RS & Opare-Obisaw C (1994) The relative validity of the repeated 24 h recall for estimating energy and selected nutrient intakes of rural Ghanaian children. Eur J Clin Nutr 48, 241–252. 8039484

[73] Pries AM , Rehman AM , Filteau S , et al. (2019) Unhealthy snack food and beverage consumption is associated with lower dietary adequacy and length-for-age z-scores among 12–23-month-olds in Kathmandu Valley, Nepal. J Nutr 149, 1843–1851. 31309223 10.1093/jn/nxz140 PMC6768809

[74] Rollo ME , Williams RL , Burrows T , et al. (2016) What are they really eating? A review on new approaches to dietary intake assessment and validation. Curr Nutr Rep 5, 307–14.

