# Supplementary material for: Accuracy and cost-effectiveness of the INDDEX24 Dietary Assessment Platform in Viet Nam
Source: Br J Nutr. 2022 May 19;129(10):1751–64. doi: 10.1017/S0007114522001507 (PMC10116183; doi:10.1017/S0007114522001507)
Supplement: Supplementary file 1 [file S0007114522001507sup001.docx]

Paper Title: Accuracy and Cost-effectiveness of the INDDEX24 Dietary Assessment Platform in Viet Nam

Authors: Jennifer Coates^1^*, Winnie Bell^1^, Peter Bakun^1^, Katherine P Adams^2^, Jérome W Somé^1,3^, Brooke Colaiezzi^1^, Ha Thi Phuong Do^4^, and Beatrice Lorge Rogers^1^

**Author Affiliations (departmental and institutional)**

^1^ Tufts University, Gerald J. and Dorothy R. Friedman School of Nutrition Science and Policy (JC, WB, PB, JWS, BC, BLR)

^2^University of California, Davis; Institue for Global Nutrition, Department of Nutrition (KPA)

^3^Institut de Recherche en Sciences de la Santé, Burkina Faso (JWS)

^4^National Institute of Nutrition, Viet Nam (HD)

***Corresponding author:**

Jennifer Coates; Tufts University Friedman School of Nutrition Science and Policy; 150 Harrison Avenue; Boston, MA 02111 USA; (617) 636-3677; Jennifer.coates@tufts.edu

Date of last update: April 7, 2022

Table of Contents: Online Supporting Material

[I. Development of Viet Nam dietary reference data for Food Matters Database 1](#_Toc100133395)

[***Food, recipe, and ingredient listing*** 1](#_Toc100133396)

[***Food composition table (FCT)*** 1](#_Toc100133397)

[***Standard recipes*** 2](#_Toc100133398)

[***Portion size estimation aid (PSEA) assignment*** 3](#_Toc100133399)

[***Portion Conversion Factors*** 3](#_Toc100133400)

[***Probes: Food tags and descriptors*** 4](#_Toc100133401)

[***Summary of dietary reference data developed as part of INDDEX24 Validation Study in Viet Nam*** 4](#_Toc100133402)

[II. Bland Altman Plots 5](#_Toc100133403)

[***Supplemental Figure A. Bland-Altman Plot for WFR-PAPI and WFR-INDDEX24: Energy*** 5](#_Toc100133404)

[***Supplemental Figure B. Bland-Altman Plot for WFR-PAPI and WFR-INDDEX24: Fat (gm)*** 5](#_Toc100133405)

[***Supplemental Figure C. Bland-Altman Plot for WFR-PAPI and WFR-INDDEX24: Protein (gm)*** 5](#_Toc100133406)

[***Supplemental Figure D. Bland-Altman Plot for WFR-PAPI and WFR-INDDEX24: Carbohydrate (gm)*** 6](#_Toc100133407)

[***Supplemental Figure E. Bland-Altman Plot for WFR-PAPI and WFR-INDDEX24: Fiber (gm)*** 6](#_Toc100133408)

[***Supplemental Figure F. Bland-Altman Plot for WFR-PAPI and WFR-INDDEX24: Vitamin A (mcg RAE)*** 6](#_Toc100133409)

[***Supplemental Figure G. Bland-Altman Plot for WFR-PAPI and WFR-INDDEX24: Vitamin C (mg)*** 7](#_Toc100133410)

[***Supplemental Figure H. Bland-Altman Plot for WFR-PAPI and WFR-INDDEX24: Calcium (mg)*** 7](#_Toc100133411)

[***Supplemental Figure I. Bland-Altman Plot for WFR-PAPI and WFR-INDDEX24I: Iron (mg)*** 7](#_Toc100133412)

[***Supplemental Figure J. Bland-Altman Plot for WFR-PAPI and WFR-INDDEX24: Zinc (mg)*** 8](#_Toc100133413)

[III. Supplemental Tables 9](#_Toc100133414)

[***Supplemental Table S1. Percent of respondents within ranges of percent error in estimating energy and nutrient intakes (24HR compared to WFR) with positive and negative errors*** 9](#_Toc100133415)

[***Supplemental Table S2. Nutrient-specific cost-effectiveness of conducting a 24HR using PAPI and INDDEX24*** 10](#_Toc100133416)

## **Development of Viet Nam dietary reference data for Food Matters Database**

### ***Food, recipe, and ingredient listing***

A master list of foods, beverages, recipes, and ingredients (henceforth referred to collectively as “food list”) was collated from a variety of data sources including the published Viet Nam Food Composition Table^[[1]](#footnote-1)^; foods from the NIN Food Photo Atlas^[[2]](#footnote-2)^; recipes and additional ingredients from the NIN book “Nutritive Values of 500 Common Dishes”,^[[3]](#footnote-3)^ which is based on household recipes reported in the 2010 General Nutrition Survey; the NIN yield factors book with simple cooked foods^[[4]](#footnote-4)^; consumption data from the INDDEX24 Feasibility Study (n=60) conducted in 2018; and recipes from common street foods in southern Viet Nam compiled in the Nutritive Value of Common Street Foods^[[5]](#footnote-5)^.

The food list elements were collated in a single spreadsheet and classified into food groups based on the Viet Nam Food Composition Table (FCT); subsequently, several food groups were sub-divided, and new groups were added to facilitate the work involved in assessing the listing for completeness. These food groupings were only used during pre-survey work. All foods were later reclassified into the FAO/WHO GIFT Food Groups (FAO/WHO).

The master food list was reviewed by a small group of experts at NIN, and for each item, a judgment was made about approximately how frequently the item would be reported in the survey in order to prioritize the foods and recipes that were most likely to need food composition information and standard recipe development. For simple cooked foods and mixed dishes, the experts also noted whether the dish was typically only prepared in homes, only prepared in restaurants/by vendors, or both. Next the experts identified which portion size estimation aids (PSEAs) were best suited for each food item and recipes, and indicated whether density factors, edible portion factors and/or yield factors were available for portion conversion calculations (see additional details below).

### ***Food composition table (FCT)***

The FCT was developed in partnership with the Viet Nam NIN Food Chemistry Department and was built on the NIN 2017 Vietnamese Food Composition Table, data from laboratory analyses conducted between 2017-2019, and other FCTs, as described below. When data were not available in the Vietnamese FCT, other FCTs were selected based on the availability of the data and the similarity of the food or country of origin. Data were borrowed from the following sources:

- ASEAN Food Composition Table, Version 2014 ^[[6]](#footnote-6)^
- Thai Food Composition Database, 2015 ^[[7]](#footnote-7)^
- FAO/INFOODS Global food composition database for pulses – version 1.0 (uPulses1.0) – 2017 ^[[8]](#footnote-8)^
- FAO/INFOODS Global food composition database for fish and shellfish – version 1.0 (uFiSh1.0) ^[[9]](#footnote-9)^
- FAO/INFOODS/TGI Global supplement database - Version 1 ^[[10]](#footnote-10)^
- Standard Tables of Food Composition in Japan, 2015 - Seventh Revised Edition^[[11]](#footnote-11)^
- *Korean Standard Food Composition Table -* 8th ed 2011^[[12]](#footnote-12)^
- USDA National Nutrient Database for Standard Reference Legacy Release, April 2018^[[13]](#footnote-13)^

The 2017 Vietnamese FCT contained only raw foods. For the purpose of the INDDEX24 validation study, the FCT was expanded to include simple cooked foods (e.g., boiled, baked, steamed). For any non-raw foods, adjustments for cooking yield and nutrient retention were made with the appropriate yield and retention factors. The final step was to check all values using FAO/INFOOD guidelines (FAO, 2012).

### ***Standard recipes***

Standard recipes for mixed dishes were calculated based on the methods described by Gibson and Ferguson (2008). This approach enables analysis at the ingredient level, which is useful for food-based analyses, amongst other things. For this method the individual raw quantity of all ingredients and the total cooked quantity are needed in order to construct an ingredient fraction. This fraction is used to derive the quantity of each individual ingredient consumed when a respondent reports consumption of a standard recipe.

Based on the master food list and the expert review (as described earlier), the priority recipes for standard recipe development were identified. This list of recipes was reviewed again, and some new recipe variations were added after considering seasonal factors that would make some foods more or less available. The final list of dietary data inputs included approximately 750 standard recipes.

Four different sub-methods were used for standard recipe development: 1) cooking method, 2) purchase method, 3) calculation method, and 4) deconstruction method, all with the primary objective of acquiring the raw amount of each ingredient and the total cooked weight. A detailed description of each approach is as follows:

- *Cooking Method:* this method was applied for deriving most of the household recipes. Each recipe was cooked one time and all raw ingredients including water, oil, salt, and seasoning, were measured before being cooked and the total quantity was weighed again right after cooking. The proportions for these recipes were based on the standard recipes presented in the NIN *Nutritive Value of 500 Common Dishes* book.
- *Purchase Method*: this method was applied for street food, particularly for those that have a (relatively) standard portion size. Three portion sizes of each recipe were purchased from three different vendors. Vendors were requested not to mix the components of the recipe. From this, the average weight of each ingredient and the total cooked weight was derived. If the ingredients were already in cooked form, then the raw/cooked ratio was applied to determine the weight in raw form.
- *Calculation Method:* this method was used in cases where information was only available on the weight of the cooked ingredient and total cooked weight of the entire recipe, so it was necessary to apply the raw/cooked ratio to get the weight of each ingredient in its raw form.
- *Deconstruction Method:* this method was applied for recipes that were variations of previously developed standard recipes. If the variation had *more* ingredients than the original standard recipe then the additional ingredients were added to the standard recipe and then total cooked weight was derived by adding the extra amount. If the variation had *fewer* ingredients, then the additional ingredients were removed from the recipe and the total cooked weight was recalculated by subtracting the weight of the ingredients that were removed.

### ***Portion size estimation aid (PSEA) assignment***

Four general types of PSEAs were used: direct weight, photos and photo substitutes, proxy measures using rice and water, and estimated weight or price of the food. All foods, recipes, and ingredients in the Global Food Matters Database were assigned two PSEAs (and, in some rare cases, three), one of which was always direct weight. The general rules that were applied to the PSEA assignment were: to use photos for foods that were discrete pieces (e.g. vegetables, fruit, meat, fish) and foods that are served in standard sizes (e.g. spring rolls, milk boxes); to use proxy rice for foods that were heaped or piled (e.g. noodles, stir fry) and proxy water for foods that were liquid (e.g. beverages and broth soups). In rare cases where none of the above were appropriate (e.g. infrequently used cuts of meat or fish), the estimated price of the raw item or estimated weight was used. As with the WFR enumerators, all 24HR enumerators used a digital scale for PSEAs that required weighing (i.e., direct weight, proxy methods).

### ***Portion Conversion Factors***

All PSEAs required specific conversion factors to allow calculation of grams consumed, with the exception of the direct weight method. Depending on the PSEA used, various conversion factors were required that were comprised of 1) density factors, 2) edible portion factors, and 3) cooking yield factors. Density factors were used with proxy rice or water, and for photo substitutes. Edible portion factors were needed for foods consumed in forms that include inedible portions (e.g., meat with bones). Cooking yield factors were used when the respondent reports a raw food (e.g., using photos of a raw food) that was consumed as cooked. The first two types of conversion factors were used in the INDDEX24 dietary reference data conversion factor table and the cooking yield factors (as well as retention factors) were accounted for in cooked foods in the FCT.

### ***Probes: Food tags and descriptors***

The INDDEX24 system uses tags and descriptors to systematize the probing process during the 24HR. A tag is the higher-level grouping (e.g., cooking method) of the descriptor (e.g., boiled, baked, stir-fried, deep fried, sautéed etc.).

### ***Summary of dietary reference data developed as part of INDDEX24 Validation Study in Viet Nam***

## **Bland Altman Plots^[[14]](#footnote-14)^***

### ***Supplemental Figure A. Bland-Altman Plot for WFR-PAPI and WFR-INDDEX24: Energy***

### ***Supplemental Figure B. Bland-Altman Plot for WFR-PAPI and WFR-INDDEX24: Fat (gm)***

### ***Supplemental Figure C. Bland-Altman Plot for WFR-PAPI and WFR-INDDEX24: Protein (gm)***

### ***Supplemental Figure D. Bland-Altman Plot for WFR-PAPI and WFR-INDDEX24: Carbohydrate (gm)***

### ***Supplemental Figure E. Bland-Altman Plot for WFR-PAPI and WFR-INDDEX24: Fiber (gm)***

### ***Supplemental Figure F. Bland-Altman Plot for WFR-PAPI and WFR-INDDEX24: Vitamin A (mcg RAE)***

### ***Supplemental Figure G. Bland-Altman Plot for WFR-PAPI and WFR-INDDEX24: Vitamin C (mg)***

### ***Supplemental Figure H. Bland-Altman Plot for WFR-PAPI and WFR-INDDEX24: Calcium (mg)***

### ***Supplemental Figure I. Bland-Altman Plot for WFR-PAPI and WFR-INDDEX24I: Iron (mg)***

### ***Supplemental Figure J. Bland-Altman Plot for WFR-PAPI and WFR-INDDEX24: Zinc (mg)***

## **Supplemental Tables**

### ***Supplemental Table S1. Percent of respondents within ranges of percent error in estimating energy and nutrient intakes (24HR compared to WFR) with positive and negative errors***

|  |  |  |  |  |  |  |  |  |  |  |  |  |
| --- | --- | --- | --- | --- | --- | --- | --- | --- | --- | --- | --- | --- |
| **PAPI Modality** | **< -50%** | **-40.1 to -50%** | **-30.1 to -40%** | **-20.1 to -30%** | **-10.1 to -20%** | **<0 to 10%** | **>0 to 10%** | **+10.1 to 20%** | **+20.1 to 30%** | **+30.1 to 40%** | **+40.1 to 50%** | **> +50%** |
| **Item count** | 0.9 | 0.0 | 1.7 | 4.3 | 12.8 | 20.5 | 20.5 | 18.0 | 10.3 | 9.4 | 1.7 | 0.0 |
| **Gram amount** | 6.8 | 5.1 | 3.4 | 7.7 | 12.0 | 18.8 | 20.5 | 14.5 | 8.6 | 2.6 | 0.0 | 0.0 |
| **Energy (kcals)** | 8.6 | 6.8 | 7.7 | 9.4 | 14.5 | 12.8 | 12.8 | 12.8 | 9.4 | 3.4 | 1.7 | 0.0 |
| **Fat (g)** | 18.8 | 5.1 | 5.1 | 5.1 | 7.7 | 9.4 | 11.1 | 11.1 | 6.0 | 7.7 | 5.1 | 7.7 |
| **Protein (g)** | 12.0 | 2.6 | 7.7 | 12.0 | 12.8 | 8.6 | 16.2 | 12.0 | 6.8 | 3.4 | 2.6 | 3.4 |
| **Carbohydrates (g)** | 5.1 | 7.7 | 9.4 | 7.7 | 18.0 | 12.0 | 15.4 | 12.0 | 7.7 | 4.3 | 0.0 | 0.9 |
| **Total Fiber (g)** | 15.4 | 5.1 | 6.8 | 1.7 | 7.7 | 13.7 | 14.5 | 9.4 | 10.3 | 6.0 | 6.0 | 3.4 |
| **Vitamin A (mcg RAE)** | 19.7 | 2.6 | 7.7 | 3.4 | 2.6 | 14.5 | 9.4 | 9.4 | 4.3 | 11.1 | 6.0 | 9.4 |
| **Vitamin C (mg)** | 24.8 | 2.6 | 5.1 | 2.6 | 6.0 | 8.6 | 8.6 | 13.7 | 7.7 | 11.1 | 6.0 | 3.4 |
| **Calcium (mg)** | 15.4 | 9.4 | 4.3 | 5.1 | 13.7 | 11.1 | 13.7 | 15.4 | 5.1 | 3.4 | 0.9 | 2.6 |
| **Iron (mg)** | 17.1 | 2.6 | 2.6 | 11.1 | 12.8 | 12.8 | 14.5 | 7.7 | 10.3 | 3.4 | 4.3 | 0.9 |
| **Zinc (mg)** | 12.8 | 10.3 | 4.3 | 7.7 | 9.4 | 12.8 | 13.7 | 12.8 | 10.3 | 3.4 | 2.6 | 0.0 |
|  |  |  |  |  |  |  |  |  |  |  |  |  |
| **INDDEX24 Modality** | **< -50%** | **-40.1 to -50%** | **-30.1 to -40%** | **-20.1 to -30%** | **-10.1 to -20%** | **<0 to 10%** | **>0 to 10%** | **+10.1 to 20%** | **+20.1 to 30%** | **+30.1 to 40%** | **+40.1 to 50%** | **> +50%** |
| **Item count** | 1.7 | 1.7 | 1.7 | 2.6 | 7.7 | 22.2 | 18.8 | 19.7 | 11.1 | 9.4 | 2.6 | 0.9 |
| **Gram amount** | 6.8 | 2.6 | 5.1 | 6.8 | 12.0 | 21.4 | 15.4 | 13.7 | 9.4 | 6.0 | 0.9 | 0.0 |
| **Energy (kcals)** | 6.0 | 6.0 | 2.6 | 8.6 | 13.7 | 14.5 | 17.1 | 13.7 | 9.4 | 5.1 | 2.6 | 0.9 |
| **Fat (g)** | 22.2 | 3.4 | 6.0 | 3.4 | 5.1 | 6.0 | 5.1 | 6.8 | 8.6 | 10.3 | 9.4 | 13.7 |
| **Protein (g)** | 8.6 | 4.3 | 6.8 | 7.7 | 7.7 | 12.0 | 12.0 | 12.8 | 9.4 | 6.8 | 4.3 | 7.7 |
| **Carbohydrates (g)** | 11.1 | 0.9 | 5.1 | 6.0 | 15.4 | 21.4 | 18.0 | 14.5 | 2.6 | 4.3 | 0.9 | 0.0 |
| **Total Fiber (g)** | 11.1 | 5.1 | 4.3 | 8.6 | 4.3 | 12.8 | 11.1 | 14.5 | 6.8 | 9.4 | 4.3 | 7.7 |
| **Vitamin A (mcg RAE)** | 12.8 | 1.7 | 5.1 | 8.6 | 12.0 | 14.5 | 12.8 | 12.0 | 8.6 | 5.1 | 4.3 | 2.6 |
| **Vitamin C (mg)** | 8.6 | 3.4 | 9.4 | 6.0 | 10.3 | 10.3 | 10.3 | 14.5 | 10.3 | 7.7 | 5.1 | 4.3 |
| **Calcium (mg)** | 13.7 | 3.4 | 6.0 | 6.8 | 8.6 | 16.2 | 11.1 | 17.1 | 6.8 | 5.1 | 3.4 | 1.7 |
| **Iron (mg)** | 19.7 | 3.4 | 2.6 | 4.3 | 7.7 | 8.6 | 4.3 | 6.8 | 12.8 | 8.6 | 11.1 | 10.3 |
| **Zinc (mg)** | 23.1 | 6.8 | 6.0 | 4.3 | 3.4 | 10.3 | 7.7 | 12.8 | 7.7 | 6.0 | 4.3 | 7.7 |

***Supplemental Table S2. Nutrient-specific cost-effectiveness of conducting a 24HR using PAPI and INDDEX24***

| Nutrient | Modality | Average percent accuracy | Cost-effectiveness^1^  (2019 USD) |
| --- | --- | --- | --- |
| Energy (kcal) | INDDEX24 | 99 | 1122 |
|  | PAPI | 93 | 1291 |
|  | Difference^2^ | 6 | -169 |
| Fat (g) | INDDEX24 | 91 | 1221 |
|  | PAPI | 96 | 1259 |
|  | Difference | -5 | -38 |
| Protein (g) | INDDEX24 | 90 | 1240 |
|  | PAPI | 95 | 1270 |
|  | Difference | -5 | -29 |
| Carbohydrate (g) | INDDEX24 | 92 | 1203 |
|  | PAPI | 93 | 1297 |
|  | Difference | -1 | -94 |
| Fiber (g) | INDDEX24 | 97 | 1141 |
|  | PAPI | 90 | 1339 |
|  | Difference | 7 | -198 |
| Vitamin A (mcg RAE) | INDDEX24 | 86 | 1284 |
|  | PAPI | 98 | 1228 |
|  | Difference | -12 | 56 |
| Vitamin C (mg) | INDDEX24 | 88 | 1266 |
|  | PAPI | 90 | 1335 |
|  | Difference | -3 | -69 |
| Calcium (mg) | INDDEX24 | 98 | 1130 |
|  | PAPI | 87 | 1379 |
|  | Difference | 11 | -249 |
| Iron (mg) | INDDEX24 | 93 | 1190 |
|  | PAPI | 91 | 1331 |
|  | Difference | 3 | -141 |
| Zinc (mg) | INDDEX24 | 95 | 1163 |
|  | PAPI | 89 | 1352 |
|  | Difference | 6 | -189 |

PAPI, pen-and-paper interviewer-administered.

^1^Cost-effectivenss was calculated as cost per average percentage point of accuracy.

^2^The difference was calculated as INDDEX24 minus PAPI. All estimates are rounded to the nearest whole number

1. National Institute of Nutrition, Ministry of Health. Vietnamese Food Composition Table. Medical Publishing House Hanoi, 2017. [↑](#footnote-ref-1)
2. National Institute of Nutrition, Ministry of Health. Food photo atlas for dietary assessment of children 2-5 years old. Medical Publishing House. Hanoi, 2014. [↑](#footnote-ref-2)
3. National Institute of Nutrition, Ministry of Health. Nutritive value of 500 common dishes. Medical Publishing House. Hanoi, 2016. [↑](#footnote-ref-3)
4. National Institute of Nutrition, Ministry of Health. Yield Factor. Medical Publishing House. Hanoi, 2017. [↑](#footnote-ref-4)
5. Ho Chi Minh City’s Nutrition Center. Nutritive value of common street foods. Medical Publishing House. Ho Chi Minh city, 2017. [↑](#footnote-ref-5)
6. Institute of Nutrition, M. U. (2014) ASEAN Food Composition Database. In, (1 ed.). Thailand. [↑](#footnote-ref-6)
7. Judprasong, Kunchit, Prapasri Puwastien, Nipa Rojroongwasinkul, Anadi Nitithamyong, Piyanut Sridonpai, Amnat Somjai. Institute of Nutrition, Mahidol University (2015). Thai Food Composition Database, Online version 2, September 2018, Thailand. <http://www.inmu.mahidol.ac.th/thaifcd>. [↑](#footnote-ref-7)
8. FAO. (2017). FAO/INFOODS Global Food Composition Database for Pulses Version 1.0 - uPulses. from FAO. [↑](#footnote-ref-8)
9. Food and Agriculture Organization of the United Nations (2016). FAO/INFOODS Global Food Composition Database for Fish and Shellfish Version 1.0- uFiSh1.0. Rome, Italy. [↑](#footnote-ref-9)
10. FAO/INFOODS/TGI Global supplement database. Retrieved from <http://www.fao.org/infoods/infoods/tables-and-databases/faoinfoods-databases/en/>. [↑](#footnote-ref-10)
11. Standard Tables of Food Composition in Japan, 2015 - Seventh Revised Edition. Retrieved from <https://www.mext.go.jp/en/policy/science_technology/policy/title01/detail01/1374030.htm>. [↑](#footnote-ref-11)
12. Korean Standard Food Composition Table - Rural Resources Development Institute - Rural Development Administration, Suwon - 8th ed 2011* (Korean with English). Retrieved from <http://koreanfood.rda.go.kr/eng/fctFoodSrchEng/engMain>. [↑](#footnote-ref-12)
13. Haytowitz, David B.; Ahuja, Jaspreet K.C.; Wu, Xianli; Somanchi, Meena; Nickle, Melissa; Nguyen, Quyen A.; Roseland, Janet M.; Williams, Juhi R.; Patterson, Kristine Y.; Li, Ying; Pehrsson, Pamela R. (2019). USDA National Nutrient Database for Standard Reference, Legacy Release. Nutrient Data Laboratory, Beltsville Human Nutrition Research Center, ARS, USDA. https://data.nal.usda.gov/dataset/usda-national-nutrient-database-standard-reference-legacy-release. Accessed 2020-08-06. [↑](#footnote-ref-13)
14. * The LOA, shown as the upper and lower limit on the plot, represent +/- 1.96 standard deviations from the mean. The bias is computed as the difference of one method minus the other method, so if one method is sometimes high and sometimes lower the average of the differences will be close to zero (i.e. with no/little bias). If the regression line is not close to zero this indicates that the two methods yield systematic errors in one direction (i.e. bias) (Bland & Altman, 1999, 2007). [↑](#footnote-ref-14)
